# Reconstruction of an Extensive Segmental Radial Shaft Bone Defect by Vascularized 3D-Printed Graft Cage

**DOI:** 10.3390/jpm14020178

**Published:** 2024-02-04

**Authors:** Philipp Mommsen, Vincent März, Nicco Krezdorn, Gökmen Aktas, Stephan Sehmisch, Peter Maria Vogt, Tobias Großner, Tarek Omar Pacha

**Affiliations:** 1 Department of Trauma Surgery, Hannover Medical School, 30625 Hannover, Germany; aktas.goekmen@mh-hannover.de (G.A.); sehmisch.stephan@mh-hannover.de (S.S.); omarpacha.tarek@mh-hannover.de (T.O.P.); 2 Department of Plastic, Aesthetic, Hand and Reconstructive Surgery, Hannover Medical School, 30625 Hannover, Germany; maerz.vincent@mh-hannover.de (V.M.); nkre@regionsjaelland.dk (N.K.); vogt.peter@mh-hannover.de (P.M.V.); 3 Department of Plastic and Breast Surgery, Roskilde University Hospital, 4000 Roskilde, Denmark; 4 BellaSeno GmbH, 04103 Leipzig, Germany; tobias.grossner@bellaseno.com; 5 BellaSeno Pty Ltd., Brisbane, QLD 4220, Australia

**Keywords:** bone reconstruction, large segmental defects, 3D-printed graft cage, vascularization

## Abstract

We report here a 46-year-old male patient with a 14 cm segmental bone defect of the radial shaft after third degree open infected fracture caused by a shrapnel injury. The patient underwent fixed-angle plate osteosynthesis and bone reconstruction of the radial shaft by a vascularized 3D-printed graft cage, including plastic coverage with a latissimus dorsi flap and an additional central vascular pedicle. Bony reconstruction of segmental defects still represents a major challenge in musculo-skeletal surgery. Thereby, 3D-printed scaffolds or graft cages display a new treatment option for bone restoration. As missing vascularization sets the limits for the treatment of large-volume bone defects by 3D-printed scaffolds, in the present case, we firstly describe the reconstruction of an extensive radial shaft bone defect by using a graft cage with additional vascularization.

## 1. Introduction

Treatment of critical-size bone defects (>5 cm) still poses numerous intriguing medical problems [1,2]. Thereby, external factors as well as aging and prolonged life expectancy in terms of demographic change result in a steady increase in post-traumatic and post-infectious bone defects [3,4]. Depending on size, location, type, and cause of bone defect, common reconstructive procedures include shortening, bone grafting (autologous, allogeneic, synthetic), the Masquelet technique, distraction osteogenesis (bone transport), and vascularized free bone transfer, in particular free vascularized fibular grafts, each having specific benefits and limitations [5,6,7,8,9,10,11,12,13]. Autologous bone grafting, the only graft material with osteogenic, osteoinductive, and osteoconductive properties, is considered the gold standard for bone regeneration [14]. However, restricted graft volume and donor-site morbidity in combination with technical innovations in tissue engineering and 3D printing have made patient-specific scaffolds an increasingly important treatment option for large bone defects. Polycaprolactone (PCL) and tricalcium phosphate (TCP) scaffolds are mainly used to provide reasonable mechanical stability, osteoconductive capabilities, and bioresorbability. Nevertheless, angiogenesis and vascularity, the fourth component of bone regeneration, represent a decisive and limiting factor for bone regeneration [15,16,17,18,19,20]. However, there is only experimental study data available dealing with vascularized tissue-engineered bone. To the authors’ knowledge, there are only limited clinical experiences on the application of vascularized graft cages for the restoration of large-volume segmental bone defects [21]. In the presented case report, we firstly describe a new surgical procedure for graft vascularization by embedding a vascular muscle arcade directly into a custom-made, 3D-printed medical-grade PCL (mPCL) scaffold as a promising treatment option for the reconstruction of extensive bone defects.

## 2. Case Report

**Figure 1:**
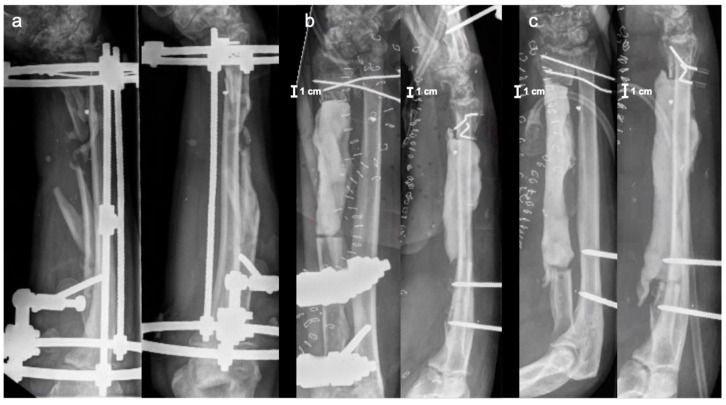
X-ray images (AP and lateral view) of the right forearm: (**a**) at the time of admission; (**b**) after 1st operation; and (**c**) before bone reconstructive surgery.

This case report presents a 46-year-old male patient who sustained a shrapnel injury during the Ukrainian war in June 2022 with traumatic thigh amputation and third degree open radial shaft defect fracture on the right side. Besides primary wound cleansing, the radial shaft fracture was stabilized by a ring fixator. Overall, ten reoperations with soft tissue and bony debridement were necessary until secondary wound closure was obtained. In December 2022, the patient was transferred to our department for the reconstruction of musculo-skeletal injuries on the right forearm. At the time of admission, the clinical examination revealed intact soft tissue coverage without macroscopic signs of infection, but with extensive scars on the palmar and dorsal forearm and a lesion of the median nerve with paresis of thumb opposition and reduced sensitivity in the dependent innervated regions of the hand. The radial and ulnar nerve, as well as peripheral blood circulation, were intact. Figure 1a shows the X-ray images of the right forearm at the time of admission.

**Figure 2:**
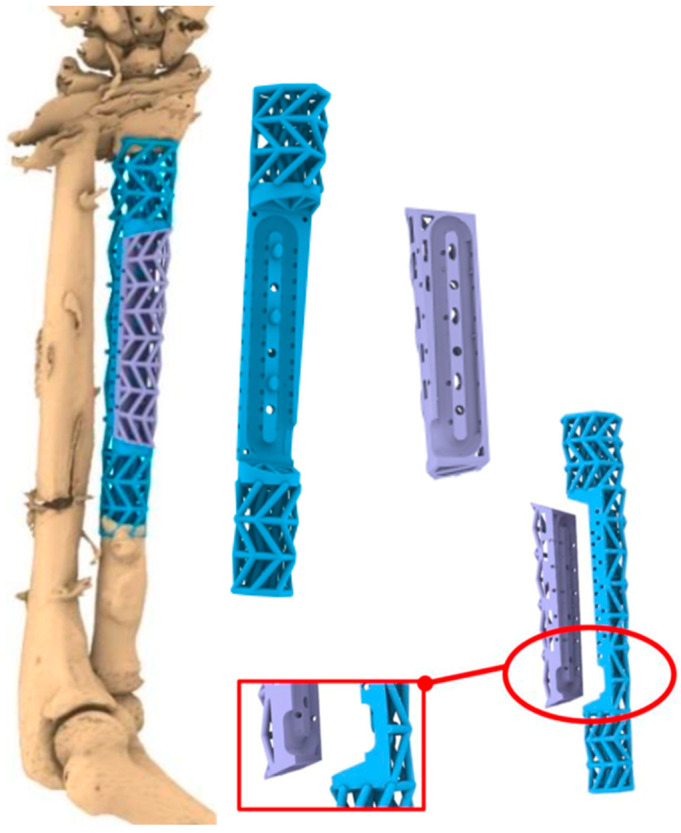
CT-based digital planning view of the scaffold with basic (blue) and locking part (purple), and outer cage aperture for graft vascularization (red frame).

**Figure 3:**
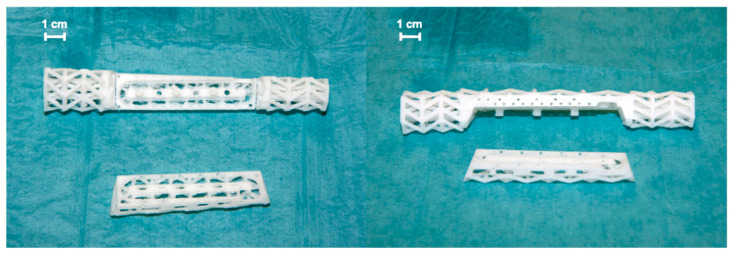
3D-printed graft cage.

In the first operation, re-debridement with resection of infected and necrotic soft and bone tissue, dead space management with antibiotic (gentamicin and vancomycin) cement spacer, and K-wire transfixation of the distal radioulnar joint were performed. Moreover, the ring fixator was replaced by conventional ulno-metacarpal external fixator (Figure 1b). The microbiological assessment showed colonization with multi-drug-resistant strains of bacteria (*Klebsiella pneumoniae*, *Corynebaterium glucoronolyticum*, *Staphylococcus haemolyticus*, *Staphylococcus epidermidis*). After five more operations and under antibiogram-adopted systemic antibiotic treatment, bacterial eradication was achieved. Wound closure was performed on the palmar forearm with a split skin graft from the ipsilateral upper arm. Systemic antibiotic therapy was continued for six more weeks after the last operation. Following an antibiotic-free time period of 4 weeks, another revision surgery with re-debridement and the exchange of cement spacer was carried out. Microbiological specimens showed no bacterial colonization. The radiological examination after this final re-debridement is shown in Figure 1c. For the proper planning of reconstructive surgery, thin-slice computer tomography (CT) of the forearm was conducted showing a 14 cm radial shaft defect. As a biological reconstruction using autologous bone was planned, a resorbable custom-made scaffold was requested from manufacturer BellaSeno GmbH (Leipzig, Germany), ensuring a secure hold of autologous bone in the large void. Based on the CT scan, segmentation of the defect was performed, followed by surgeon-driven individual design of a 90% open porous scaffold structure perfectly fitting into the void according to the patient’s individual anatomy. To ensure a proper internal vascularization of the scaffold, a groove was requested as a design feature for the future positioning of an arterio-venous loop (AV-loop) or a central vascular pedicle during reconstructive surgery. After approval and design freeze by the prescriber, the scaffold was 3D-printed (FDM, additive manufactured) as a patient-specific custom-made graft cage, fabricated (BellaSeno GmbH, Leipzig, Germany) for biological bony defect restoration. The defect volume was calculated with ~44 mL. The manufactured scaffold is completely bioresorbable, consisting of 100% medical-grade polycaprolactone (mPCL) and provides osteoconductive properties, making additional autologous bone grafting and/or synthetic bone substitutes mandatory. It consists of an inner and outer support frame with a basic and a locking part. The scaffold base maximum dimensions are at a maximal depth of ~21.8 mm, maximal width of ~23.7 mm, and maximal height of ~152.2 mm. The scaffold lid maximum dimensions are a maximal depth of ~12.4 mm, maximal width of ~19.6 mm, and maximal height of ~85.2 mm. The overall scaffold porosity is 73%, and the scaffold overall weight ~13.3 g ± 5%. Biomechanically, the scaffold withstands a 3 mm deformation under 90 N. Consequently, the scaffold is not a load-bearing structure. Therefore, the graft cage is intended to be used in conjunction with rigid osteosynthesis only. For graft vascularization by placing an AV-loop or a central vascular pedicle, an aperture and groove located on the outer cage were provided as a specific design request to the manufacturer. The diameter of the aperture for the AV-loop or vascular pedicle is 5 mm. The volume of the embedded loop or vascular pedicle was calculated with ~1.5 mL. The CT-based digital planning view and 3D-printed graft cage are shown in Figure 2 and Figure 3, respectively.

**Figure 4:**
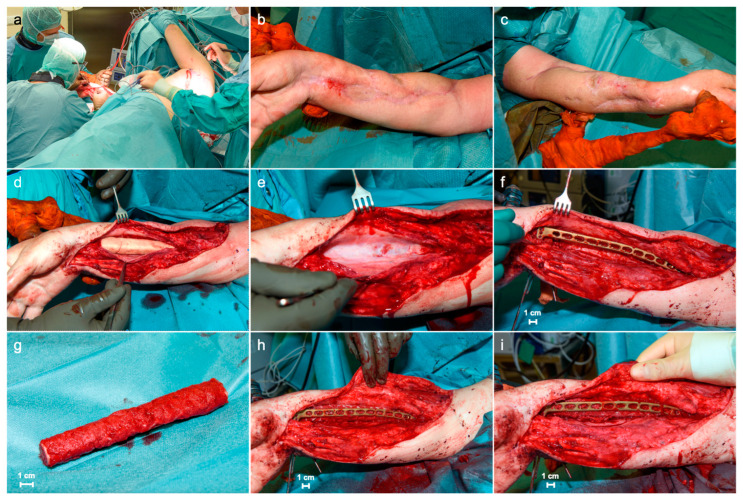
Intraoperative images: (**a**) patient’s positioning and setting; (**b**) palmar and (**c**) dorsal soft tissues of the right forearm; (**d**) palmar approach to the forearm and preparation of the cement spacer; (**e**) Masquelet membrane; (**f**) palmar fixed-angle plate osteosynthesis of the radial shaft; (**g**) scaffold filled up with autologous bone graft; (**h**,**i**) showing the surgical site after graft insertion.

In October 2023, open reduction and internal fixation, as well as bone reconstruction of the radial shaft, were performed using a conventional 3.5 mm LCP volar dia-metaphyseal distal radius plate (DePuy Synthes, Zuchwil, Switzerland) and the aforementioned vascularized graft cage (BellaSeno GmbH, Leipzig, Germany). The scaffold perfectly fit into the void without any need for further adjustment. Additionally, soft tissue coverage was made by a latissimus dorsi flap from the left side. Cement spacer removal, plate osteosynthesis, and graft insertion were made using a palmar approach to the right forearm (Figure 4). Prior to insertion, the scaffold was filled up with 35 cc autologous bone graft taken from the left femur with the reamer irrigator aspirator system (RIA 2 system, DePuy Synthes, Zuchwil, Switzerland) as shown in Figure 4.

**Figure 5:**
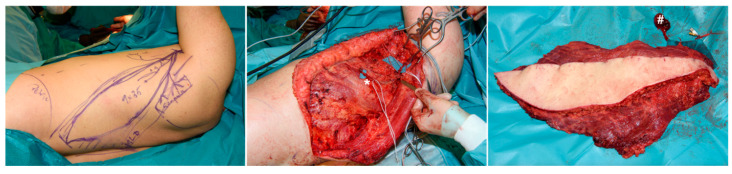
Preparation of left-sided latissimus dorsi flap, including vascular serratus arcade (*) and small portion of serratus anterior muscle (#).

For soft tissue coverage, a latissimus dorsi flap was prepared on the left side with an additional vascular serratus arcade, including a small portion of serratus anterior muscle (Figure 5).

**Figure 6:**
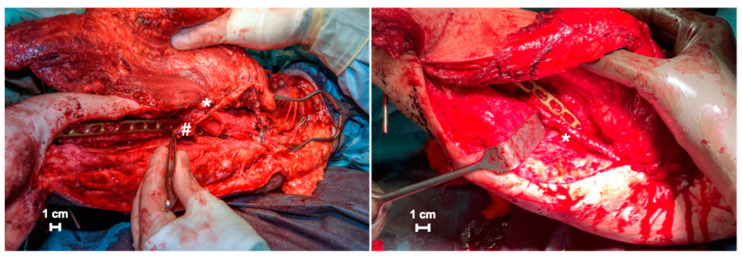
Graft vascularization by embedding vascular pedicle into the scaffold (* vascular serratus arcade; # small portion of serratus anterior muscle).

For graft cage vascularization, this vascular pedicle was placed and fixed into the aperture located on the outer cage frame (Figure 6).

**Figure 7:**
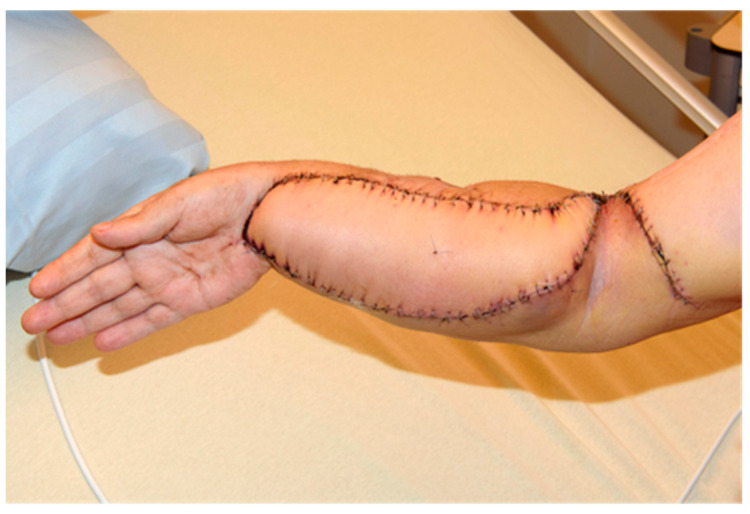
Patient’s right forearm after surgical intervention.

**Figure 8:**
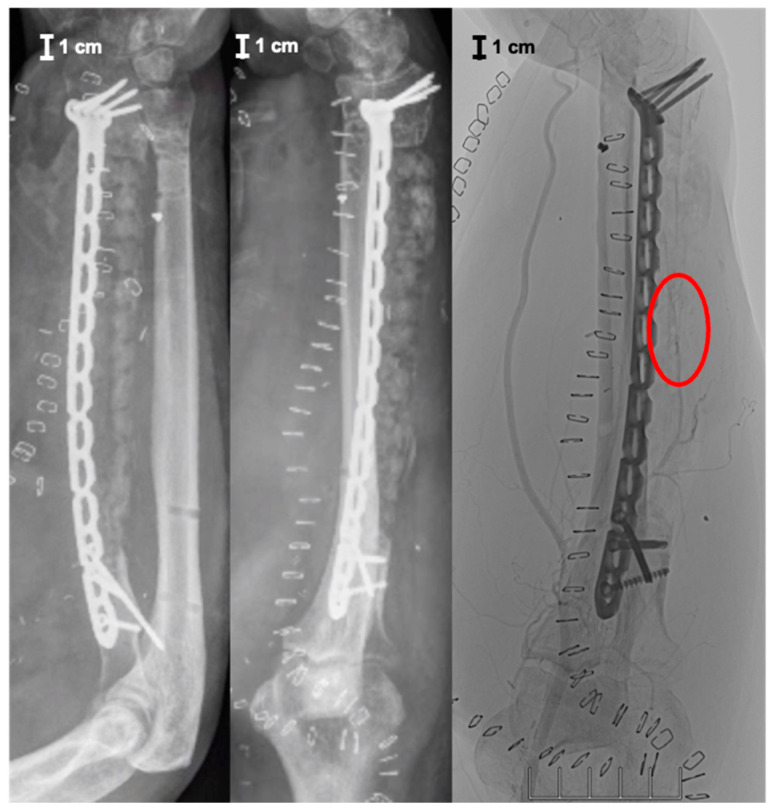
Postoperative X-ray images (AP and lateral view) and digital subtraction angiography (DSA) of the right forearm showing adequate implant position and graft vascularization (red frame).

**Figure 9:**
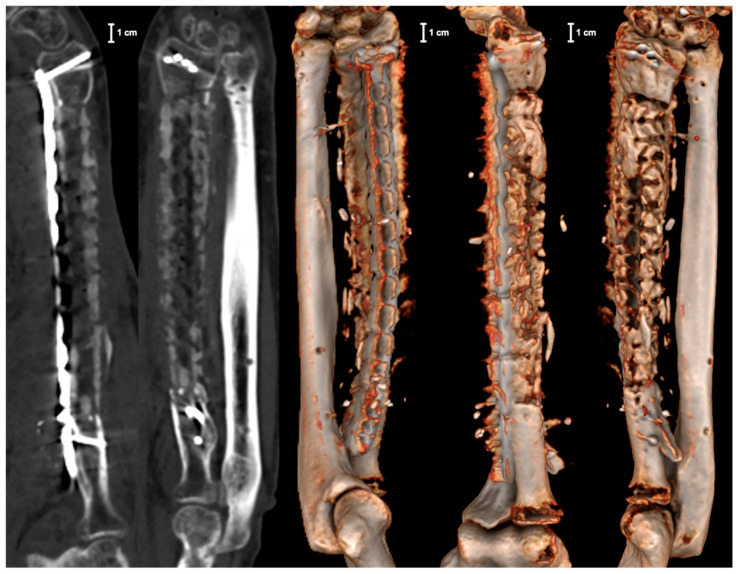
Postoperative computer tomography (CT) of the right forearm.

The flap itself was connected to brachial vessels by end-to-side anastomoses. The clinical result, postoperative X-ray, digital subtraction angiography, and CT scan showing an accurate implant position with proper articulation and adequate graft vascularization are displayed in Figure 7, Figure 8 and Figure 9.

**Figure 10:**
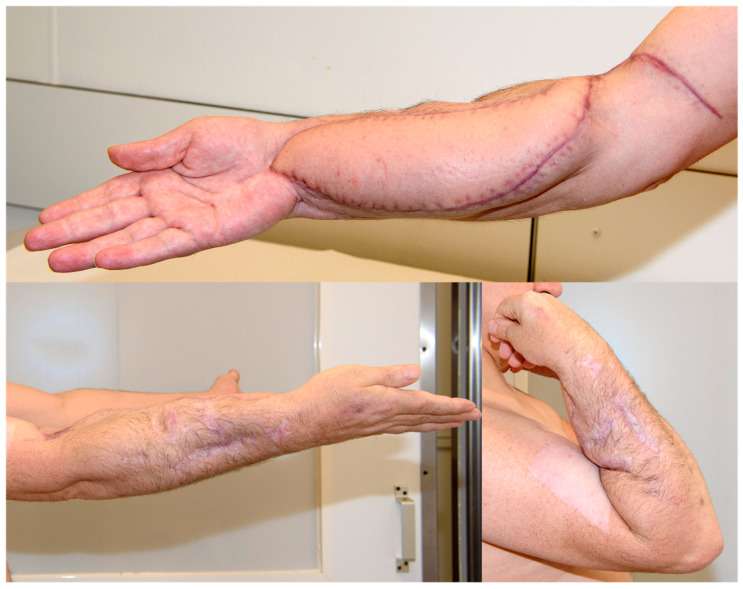
Patient’s right forearm and elbow function 3 months after surgical intervention.

**Figure 11:**
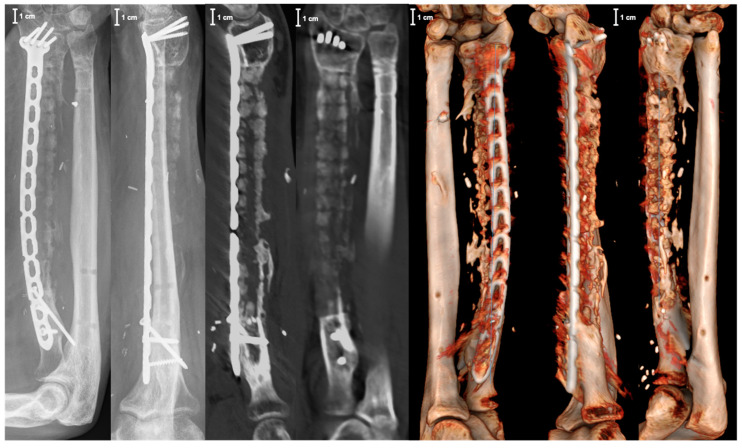
X-ray images (AP and lateral view) and computer tomography (CT) of the right forearm 3 months after surgical intervention.

Microbiological specimens taken during this operation still remained without bacterial colonization. However, systemic antibiotic therapy was applied for another two weeks postoperatively. At 3 months follow-up, no clinical signs of infection were apparent, with an adequate elbow function (Figure 10). The radiological diagnostics, including X-ray and CT scan at this timepoint, showed no implant failure and timely bony integration, especially in the interface between the distal and proximal host bone and the graft cage (Figure 11).

## 3. Discussion

Reconstruction of large segmental bone defects still represents a major challenge in musculo-skeletal surgery. Patient-specific scaffolds represent a new treatment option for bony defect restoration [22,23,24]. Thereby, 3D printing based on a CT scan enables individualized and tailor-made fitting of the custom-made scaffold into the bone defect. In the present case, we used a vascularized graft cage (BellaSeno GmbH, Leipzig, Germany). This fully bioresorbable 3D-printed scaffold consists solely of polycaprolactone and provides osteoconductive characteristics. Accordingly, additional autologous bone grafting and/or synthetic bone substitutes offering osteogenic and osteoinductive properties are imperative. In the present case, a sufficient amount of autologous bone graft was harvested from the contralateral femur using the RIA system, helping to reduce donor-site morbidity [25,26]. Besides osteogenesis, osteoconductivity, and osteoinductivity, the induction of angiogenesis and vascularity play a crucial role after scaffold implantation [15,16,17,18,19,20]. In this context, the impregnation of 3D-printed scaffolds with angiogenetic factors and/or cells represents a promising concept in tissue engineering [27,28]. So far, there is only experimental study data available dealing with vascularized tissue-engineered bone. In the present case, graft cage vascularization was achieved by embedding perfused capillary muscle tissue into a patient-specific, 3D-printed scaffold in combination with a freedom latissimus dorsi flap for soft tissue coverage. In the case of a missing vascular muscle arcade, placing an arterio-venous loop into the scaffold would have been an alternative option. However, preparing an AV-loop is surgically more sophisticated as micro-anastomoses are needed and a microcapillary environment like in a natural pedicle is missing. To the authors’ knowledge, this is the first description globally of such a surgical technique for graft vascularization. Therefore, we primarily focused on the feasibility and safety of the presented surgical procedure. However, X-ray images of the right forearm 3 months after the surgical procedure showed a timely osseous integration, yet without full bone defect restoration. The next appointment with clinical and radiological examination is intended 6 months after the surgical intervention. Additional long-term follow-up is planned for 12, 18, and 24 months after the operation, including X-ray and an optional CT scan for evaluating further bony consolidation. It might be argued that free vascularized fibular grafts are more commonly used compared to custom-made graft cages offering excellent results with a bony defect restoration rate of 95% [29]. However, several disadvantages of free vascularized fibular grafts have been reported. Donor-site morbidity, sophisticated microsurgical technique, long osseus consolidation time, and fragile bone reconstruction due to limited transplant diameter represent major problems [30]. Additionally, different postoperative complications are reported in up to 54%, including partial flap necrosis, arterial thrombosis, venous congestion, and graft fracture [29]. In comparison to the Masquelet technique, the use of patient-specific, 3D-printed scaffolds is associated with a lower bone graft volume needed for defect reconstruction. Furthermore, the scaffold’s support frame prevents the sedimentation and diffusion effects of autologous bone graft and results in tubular bone formation with favorable biomechanical properties compared to unphysiological rigid bone block in the Masquelet technique [31,32,33,34]. Furthermore, central perfusion problems with an increased risk for graft resorption can occur with pure bone grafting in critical-size segmental bone defects > 3–5 cm [35]. If this threshold is exceeded, transplant and graft vascularization are recommended [1,36]. In the present case report, graft vascularization was achieved by a vascular pedicle placed and fixed into the outer cage frame more in the middle part of the scaffold, as indicated by the red frame in Figure 8. This might be unfavorable as the most critical bone healing areas are located in the interface between the distal and proximal host bone and the graft cage. However, in the present case, only one vascular pedicle consisting of a vascular serratus arcade and a small portion of serratus anterior muscle was available. The positioning and embedding of this vascular pedicle into the scaffold were mainly limited by its length. In order to avoid any torsion, stretching, and kinking, the vascular pedicle entered the scaffold in a flat entrance angle through an outer cage aperture at the proximal third of the graft cage and finally ended in the middle part. In addition, it has to be mentioned that there are some challenges with regard to regulatory requirements as the custom-made scaffold used in the present case is not an officially certified medicinal product. In fact, the surgical procedure described represents an individual medical treatment of the surgeon’s own responsibility. The patient must be fully informed about the healing attempt, and an additional consent form has to be signed. However, the custom-made polycaprolactone bone scaffold (MDR 770239) aligns with the stringent criteria defined for ‘custom-made devices’ in EU regulation 2017/745 article 2(3) as it is specifically made in accordance with a written prescription of any person authorized by national law by virtue of that person’s professional qualifications, has specific design characteristics, and is intended for the sole use of a particular patient exclusively to meet their individual conditions and needs. Additionally, it is not mass-produced, and no templates, precursors, or base product have been made to be adapted later. Moreover, adherence to regulatory standards involves compliance with EU regulation 2017/745 article 52(8) and annex XIII. Notably, the commitment to quality is underscored by the possession of an EU quality management system certificate, in accordance with regulation (EU) 2017/745 and annex IX chapter I and III. This certification solidifies the custom-made polycaprolactone bone scaffold as a class III implantable medical device, attesting to its adherence to the highest standards of safety and efficacy.

In general, 3D-printed scaffolds represent a promising approach in order to overcome the limitations, morbidities, and complications of the conventional treatment options for critical-size bone defects like acute shortening, amputation, bone grafting, the Masquelet technique, bone transport, and vascularized free bone transfer. Firstly, 3D-printed titanium scaffolds were used mainly in complex cases with bone defects at the distal tibia and foot. The study results indicate sufficient implant strength and stability with acceptable clinical outcomes [37,38]. However, difficulties in bone formation visualization within titanium scaffolds as well as wear and corrosion problems with potential chronic inflammation display major limitations [37,38,39]. Furthermore, differences in elasticity between titanium and natural bone may lead to stress shielding and bone resorption [40]. Accordingly, custom-made biodegradable scaffolds were developed [41]. Thereby, medical-grade polycaprolactone (mPCL) in combination with tricalcium phosphate (TCP) was identified as a suitable biomaterial with adequate mechanical stability, osteoconduction, and bioresorbability for the restoration of critical-size bone defects [22,42,43]. The current case series described complete bone repair and osseous remodeling in large femoral and tibial bone defects using such 3D-printed scaffolds [23,24]. Recently, angiogenesis and vascularity came into focus as decisive and limiting factors for bone regeneration [15,16,17,18,19,20]. In addition, the impregnation of 3D-printed graft cages with angiogenetic factors and/or cells and the use of vascularized corticoperiosteal–cutaneous flaps were achieved [22,44,45]. In 1993, Cappana et al. first described a surgical procedure for graft vascularization in terms of a “combined graft” [46]. In detail, this surgical technique is based on the idea of bridging large bone defects by a massive allograft as an outer peripheral shell with an additional centrally placed micro-vascular fibular autograft [46]. Castrisos et al. developed a modified Capanna technique and reported four cases of bone defect reconstruction in 2022 [47]. Besides one congenital bone defect, bone defects have resulted from osteomyelitis, trauma, and bone tumor resection (Ewing sarcoma) [47]. Bone defect reconstruction was achieved by using 3D-printed mPCL-TCP scaffolds wrapped in vascularized free cortico-periosteal flaps [47]. In the presented case report, we firstly describe a new surgical technique for graft vascularization by embedding a vascular muscle arcade directly into a patient-specific, 3D-printed mPCL scaffold. The described surgical procedure represents an innovative and promising approach for the restoration of extensive bone defects, avoiding the need for cortico-periosteal flaps or centrally placed micro-vascular fibular autografts.

## Data Availability

No new data were created or analyzed in this study. Data sharing is not applicable to this article.

## References

[1] Huang Q., Xu Y.B., Ren C., Li M., Zhang C.C., Liu L., Wang Q., Lu Y., Lin H., Li Z. (2022). Bone transport combined with bone graft and internal fixation versus simple bone transport in the treatment of large bone defects of lower limbs after trauma. BMC Musculoskelet. Disord..

[2] Wang P., Wu Y., Rui Y., Wang J., Liu J., Ma Y. (2021). Masquelet technique for reconstructing bone defects in open lower limb fracture: Analysis of the relationship between bone defect and bone graft. Injury.

[3] Kazmirchuk A., Yarmoliuk Y., Lurin I., Gybalo R., Burianov O., Derkach S., Karpenko K. (2022). Ukraine’s Experience with Management of Combat Casualties Using NATO’s Four-Tier “Changing as Needed” Healthcare System. World J. Surg..

[4] Cillóniz C., Rodríguez-Hurtado D., Torres A. (2018). Characteristics and Management of Community-Acquired Pneumonia in the Era of Global Aging. Med. Sci..

[5] Friedrich J.B., Moran S.L., Bishop A.T., Shin A.Y. (2009). Free vascularized fibula grafts for salvage of failed oncologic long bone reconstruction and pathologic fractures. Microsurgery.

[6] Pedersen W.C., Person D.W. (2007). Long bone reconstruction with vascularized bone grafts. Orthop. Clin. N. Am..

[7] Ghert M., Colterjohn N., Manfrini M. (2007). The use of free vascularized fibular grafts in skeletal reconstruction for bone tumors in children. J. Acad. Orthop. Surg..

[8] Kong L.-C., Li H.A., Kang Q.-L., Li G. (2020). An update to the advances in understanding distraction histogenesis: From biological mechanisms to novel clinical applications. J. Orthop. Translat..

[9] Dugan T.R., Hubert M.G., Siska P.A., Pape H.-C., Tarkin I.S. (2013). Open supracondylar femur fractures with bone loss in the polytraumatized patient—Timing is everything!. Injury.

[10] Catagni M.A., Azzam W., Guerreschi F., Lovisetti L., Poli P., Khan M.S., Di Giacomo L.M. (2019). Trifocal versus bifocal bone transport in treatment of long segmental tibial bone defects. Bone Jt. J..

[11] Fuchs T., Stolberg-Stolberg J., Michel P.A., Garcia P., Amler S., Wähnert D., Raschke M.J. (2021). Effect of Bone Morphogenetic Protein-2 in the Treatment of Long Bone Non-Unions. J. Clin. Med..

[12] Gardner M.P., Beason A.M. (2021). Plate-Assisted Bone Segment Transport Versus Precice Bone Transport Nail. J. Orthop. Trauma.

[13] Valtanen R.S., Yang Y.P., Gurtner G.C., Maloney W.J., Lowenberg D.W. (2021). Synthetic and Bone tissue engineering graft substitutes: What is the future?. Injury.

[14] Schmidt A.H. (2021). Autologous bone graft: Is it still the gold standard?. Injury.

[15] Yazdanpanah Z., Johnston J.D., Cooper D.M.L., Chen X. (2022). 3D Bioprinted Scaffolds for Bone Tissue Engineering: State-Of-The-Art and Emerging Technologies. Front. Bioeng. Biotechnol..

[16] Leach J.K., Kaigler D., Wang Z., Krebsbach P.H., Mooney D.J. (2006). Coating of VEGF-releasing scaffolds with bioactive glass for angiogenesis and bone regeneration. Biomaterials.

[17] Kusumbe A.P., Ramasamy S.K., Adams R.H. (2014). Coupling of angiogenesis and osteogenesis by a specific vessel subtype in bone. Nature.

[18] Anada T., Pan C.-C., Stahl A.M., Mori S., Fukuda J., Suzuki O., Yang Y. (2019). Vascularized Bone-Mimetic Hydrogel Constructs by 3D Bioprinting to Promote Osteogenesis and Angiogenesis. Int. J. Mol. Sci..

[19] Klenke F.M., Liu Y., Yuan H., Hunziker E.B., Siebenrock K.A., Hofstetter W. (2008). Impact of pore size on the vascularization and osseointegration of ceramic bone substitutes in vivo. J. Biomed. Mater. Res. A.

[20] Ramasamy S.K., Kusumbe A.P., Wang L., Adams R.H. (2014). Endothelial Notch activity promotes angiogenesis and osteogenesis in bone. Nature.

[21] Laubach M., Hildebrand F., Suresh S., Wagels M., Kobbe P., Gilbert F., Kneser U., Holzapfel B.M., Hutmacher D.W. (2023). The Concept of Scaffold-Guided Bone Regeneration for the Treatment of Long Bone Defects: Current Clinical Application and Future Perspective. J. Funct. Biomater..

[22] Henkel J., Medeiros Savi F., Berner A., Fountain S., Saifzadeh S., Steck R., Epari D.R., Woodruff M.A., Knackstedt M., Schuetz M.A. (2021). Scaffold-guided bone regeneration in large volume tibial segmental defects. Bone.

[23] Kobbe P., Laubach M., Hutmacher D.W., Alabdulrahman H., Sellei R.M., Hildebrand F. (2020). Convergence of scaffold-guided bone regeneration and RIA bone grafting for the treatment of a critical-sized bone defect of the femoral shaft. Eur. J. Med. Res..

[24] Laubach M., Suresh S., Herath B., Wille M.-L., Delbrück H., Alabdulrahman H., Hutmacher D.W., Hildebrand F. (2022). Clinical translation of a patient-specific scaffold-guided bone regeneration concept in four cases with large long bone defects. J. Orthop. Translat..

[25] Dawson J., Kiner D., Gardner W., Swafford R., Nowotarski P.J. (2014). The reamer-irrigator-aspirator as a device for harvesting bone graft compared with iliac crest bone graft: Union rates and complications. J. Orthop. Trauma.

[26] Dimitriou R., Mataliotakis G.I., Angoules A.G., Kanakaris N.K., Giannoudis P.V. (2011). Complications following autologous bone graft harvesting from the iliac crest and using the RIA: A systematic review. Injury.

[27] Wang H., Li X., Lai S., Cao Q., Liu Y., Li J., Zhu X., Fu W., Zhang X. (2023). Construction of Vascularized Tissue Engineered Bone with nHA-Coated BCP Bioceramics Loaded with Peripheral Blood-Derived MSC and EPC to Repair Large Segmental Femoral Bone Defect. ACS Appl. Mater. Interfaces.

[28] Xu J., Shen J., Sun Y., Wu T., Sun Y., Chai Y., Kang Q., Rui B., Li G. (2022). In vivo prevascularization strategy enhances neovascularization of β-tricalcium phosphate scaffolds in bone regeneration. J. Orthop. Translat..

[29] Arai K., Toh S., Tsubo K., Nishikawa S., Narita S., Miura H. (2002). Complications of vascularized fibula graft for reconstruction of long bones. Plast. Reconstr. Surg..

[30] Lenze U., Pohlig F., Knebel C., Lenze F., Harrasser N., Mühlhofer H., Toepfer A., Rechl H., von Eisenhart-Rothe R. (2017). Autologous fibula transplantation for reconstruction of bone defects. Orthopäde.

[31] Miska M., Schmidmaier G. (2020). Diamond concept for treatment of nonunions and bone defects. Unfallchirurg.

[32] Sen M.K., Miclau T. (2007). Autologous iliac crest bone graft: Should it still be the gold standard for treating nonunions?. Injury.

[33] Oliva F., Migliorini F., Cuozzo F., Torsiello E., Hildebrand F., Maffulli N. (2021). Outcomes and complications of the reamer irrigator aspirator versus traditional iliac crest bone graft harvesting: A systematic review and meta-analysis. J. Orthop. Traumatol..

[34] Fischer C., Mendel T., Hückstädt M., Hofmann G.O., Klauke F. (2023). Reconstruction of a metadiaphyseal bone defect after open comminuted fracture of the proximal femur using a modified Masquelet technique. Unfallchirurgie.

[35] Weiland A.J., Phillips T.W., Randolph M.A. (1984). Bone grafts: A radiologic, histologic, and biomechanical model comparing autografts, allografts, and free vascularized bone grafts. Plast. Reconstr. Surg..

[36] Hierner R., Täger G., Nast-Kolb D. (2009). Vascularized bone transfer. Unfallchirurg.

[37] Tetsworth K., Woloszyk A., Glatt V. (2019). 3D printed titanium cages combined with the Masquelet technique for the reconstruction of segmental femoral defects: Preliminary clinical results and molecular analysis of the biological activity of human-induced membranes. OTA Int..

[38] Gamieldien H., Ferreira N., Birkholtz F.F., Hilton T., Campbell N., Laubscher M. (2022). Filling the gap: A series of 3D-printed titanium truss cages for the management of large, lower limb bone defects in a developing country setting. Eur. J. Orthop. Surg. Traumatol..

[39] Jia Z., Xu X., Zhu D., Zheng Y. (2023). Design, printing, and engineering of regenerative biomaterials for personalized bone healthcare. Prog. Mater. Sci..

[40] Herzog D., Seyda V., Wycisk E., Emmelmann C. (2016). Additive manufacturing of metals. Acta Mater..

[41] Hutmacher D.W. (2000). Scaffolds in tissue engineering bone and cartilage. Biomaterials.

[42] Sparks D.S., Medeiros Savi F., Saifzadeh S., Wille M.-L., Wagels M., Hutmacher D.W. (2022). Bone regeneration exploiting corticoperiosteal tissue transfer for scaffold-guided bone regeneration. Tissue Eng. Part C Methods.

[43] Sparks D.S., Saifzadeh S., Medeiros Savi F., Dlaska C.E., Berner A., Henkel J., Reichert J.C., Wullschleger M., Ren J., Cipitria A. (2020). A preclinical large-animal model for the assessment of critical-size load-bearing bone defect reconstruction. Nat. Protoc..

[44] Sparks D.S., Savi F.M., Dlaska C.E., Saifzadeh S., Brierly G., Ren E., Cipitria A., Reichert J.C., Wille M.-L., Schuetz M.A. (2020). Convergence of scaffold-guided bone reconstruction and surgical vascularization strategies-a quest for regenerative matching axial vascularization. Front. Bioeng. Biotechnol..

[45] Sparks D.S., Savi F.M., Dlaska C.E., Saifzadeh S., Brierly G., Ren E., Cipitria A., Reichert J.C., Wille M.-L., Schuetz M.A. (2023). Convergence of scaffold-guided bone regeneration principles and microvascular tissue transfer surgery. Sci. Adv..

[46] Capanna R., Bufalini C., Campanacci M. (1993). A new technique for reconstructions of large metadiaphyseal bone defects. Orthop. Traumatol..

[47] Castrisos G., Gonzalez Matheus I., Sparks D., Lowe M., Ward N., Sehu M., Wille M.-L., Phua Y., Medeiros Savi F., Hutmacher D. (2022). Regenerative matching axial vascularisation of absorbable 3D-printed scaffold for large bone defects: A first in human series. J. Plast. Reconstr. Aesthet. Surg..

